# Association of Breastfeeding Duration with Neurodevelopmental Outcomes in an Enriched Familial Likelihood Cohort for Autism Spectrum Disorder

**DOI:** 10.1007/s10578-024-01700-7

**Published:** 2024-04-24

**Authors:** Ruchi Punatar, Kathleen Angkustsiri, Laura R. Kair, Daniel J. Tancredi, Danielle J. Harvey, Rebecca J. Schmidt

**Affiliations:** 1 Division of Developmental Behavioral Pediatrics, Department of Pediatrics, University of California Davis, Sacramento, CA USA; 2 UC Davis MIND (Medical Investigation of Neurodevelopmental Disorders) Institute, University of California Davis, Sacramento, CA USA; 3 Department of Pediatrics, University of California Davis, Sacramento, CA USA; 4 Department of Public Health Sciences, University of California Davis, One Shields Avenue, Med Sci 1C, Davis, CA 95616 USA

**Keywords:** Autism Spectrum Disorder, ASD, Breastfeeding, Enriched-Likelihood Cohort, Cognitive ability, MARBLES

## Abstract

This study aimed to compare the breastfeeding (BF) duration of the younger siblings of children with ASD in an enriched-likelihood cohort for autism spectrum disorder (ASD), and to determine whether longer BF duration was associated with differences in neurodevelopmental outcomes in this cohort. Information on BF practices was collected via surveys in the MARBLES (Markers of Autism Risk in Babies-Learning Early Signs) study. Developmental evaluations, including the Mullen Scales of Early Learning and the Autism Diagnostic Observation Schedule, were conducted by expert clinicians. Participants’ neurodevelopmental outcome was classified by an algorithm into three groups: typical development, ASD, and non-typical development. The median duration of BF was 10.70 months (interquartile range of 12.07 months). There were no significant differences in the distribution of duration of BF among the three neurodevelopmental outcome categories. Children in this enriched-likelihood cohort who were breastfed for > 12 months had significantly higher scores on cognitive testing compared to those who were breastfed for 0–3 months. There was no significant difference in ASD symptomatology or ASD risk based on BF duration.

**Supplementary Information:**

The online version contains supplementary material available at 10.1007/s10578-024-01700-7.

## Introduction

Autism spectrum disorder (ASD) is a neurodevelopmental condition characterized by persistent deficits in social communication and social interaction, along with restricted and repetitive pattern of behaviors or interests [2]. Behavioral signs of ASD are not thought to be present at birth, but emerge in early childhood [37]. A combination of both genetic and environmental factors is believed to contribute to ASD risk [19, 32]. With the current prevalence of ASD in the United States at 1 out of 36 children [31], there is growing interest to identify risk and protective factors. Breastfeeding (BF) has been suggested as a potentially modifiable protective factor that may decrease the risk of ASD [17, 48] or autistic traits [8].

BF is a dynamic, bidirectional social behavior designed to transfer nutrients and build psychosocial bond between the parent and infant [42]. The World Health Organization (WHO) defines BF as when an infant receives breast milk, including expressed milk or from a wet nurse [52]. Multiple health organizations, including the American Academy of Pediatrics and the WHO, recommend exclusive BF for 6 months and then continued BF along with complementary food for as long as mutually desired by the mother and child for 2 years or beyond [33, 53]. Breast milk is believed to be the ideal nutrition for most infants since it has evolved to be finely attuned to the requirements of an infant [4].

Multiple studies have shown that BF is associated with improved cognitive development [3, 6, 28]. While the role of confounders, such as maternal IQ or home environment, are thought to be potential explanations for this relationship [13, 54], systematic reviews and meta-analyses continue to suggest some cognitive advantage attributed to BF even after adjustment for confounders [21, 50]. Horta et al. [21] found that breastfed subjects achieved a higher IQ with a mean difference of 3.44 points (95% confidence interval: 2.30, 4.58), and when controlling for maternal IQ, the mean difference was smaller at 2.62 points (95% confidence interval: 1.25; 3.98).

Similarly, researchers have suggested that there could be an association between BF and other areas of neurodevelopment, such as ASD. An early study suggested weaning BF in the first week of life was associated with infantile autism [47]. More recent studies have also found that no BF or partial BF is associated with increased odds of an ASD diagnosis [23, 43]. Additionally, children with ASD were found to be more likely to have experienced a shorter duration of BF [16, 29]. Soke et al. [45] found no significant difference in percentages for initiation of BF for children diagnosed with ASD and the control group, but shorter duration of BF for children with ASD. Similarly, longer duration of BF has been associated with a decline in risk of ASD [1, 7, 44] and fewer autistic traits [8]. Meta-analyses evaluating this association have suggested that children with ASD were significantly less likely to have been breastfed than children without ASD [48] and that exclusive BF and longer duration of BF are associated with lower ASD risk [17].

In contrast, a study using data from the National Survey of Children's Health found that ASD diagnosis was not associated with any BF, exclusive BF for 3 or 6 months, nor duration of BF [24]. Additional studies found that when children with pervasive developmental disorder [10] or ASD [15, 35] were compared to a control group, there were no differences in BF rates.

There are multiple biological reasons that could explain an association between BF and protection against ASD risk. Breast milk has constituents, such as long chain polysaturated fatty acids and oxytocin, which influence brain development and maturation [11, 27]. BF also promotes parent–child bonding which can impact social behavior [26]. Additionally, breast milk contributes to the development of the gut microbiome. Early life microbial dysbiosis could impact neurodevelopment [46] and play a role in the interface between the environmental and genetic risk factors of ASD [51].

The existing research on the association between BF and ASD has some limitations. Many of the studies might have been influenced by recall bias due to the retrospective nature of the study [1, 44]; others used variable criteria for ASD, such as autistic traits [8] or parent report of ASD diagnosis [43]. Furthermore, to our knowledge, none of the studies prospectively evaluated the role of BF in children at increased risk for ASD, such as a sibling of a child with ASD. This population is important to study since families who have one child with ASD may be interested in ways to decrease the risk in subsequent children, especially since the estimated recurrence risk of ASD in affected families is close to 19% [40].

A prospective enriched-likelihood cohort design study is ideal to evaluate the association between BF and ASD. The prospective design allows for obtaining accurate exposure information from the time period prior to onset of symptoms. The enriched-likelihood design for conditions such as ASD, with relatively low prevalence, allows for increased efficiency when following a cohort by ensuring a higher proportion of the outcome of interest will be present.

The objective of this study was to evaluate the BF practices of an enriched-likelihood cohort for ASD and to examine whether neurodevelopmental outcomes are associated with BF in this population. We hypothesized that for our enriched-likelihood cohort, longer duration of BF would be associated with improved scores on standardized neurodevelopmental assessments and lower risk for ASD.

### Hypotheses

#### Hypothesis 1:

For children in an enriched-likelihood cohort, BF for a longer duration will be associated with higher scores on the Mullen Scales of Early Learning. For children in an enriched-likelihood cohort, BF for a longer duration will be associated with lower Autism Diagnostic Observation Schedule comparison scores.

#### Hypothesis 2

For children in an enriched-likelihood cohort, BF for longer duration will be associated with a lower risk for ASD.

## Methods

### Study Population

Data were collected in the MARBLES (Markers of Autism Risk in Babies-Learning Early Signs) study, a prospective observational study with an enriched-likelihood cohort design. The aim of the study was to identify early biological and environmental markers associated with increased risk for ASD. The study began enrolling in 2006 and is still ongoing [20].

For MARBLES recruitment, families with 1 or more children with ASD were contacted during a pregnancy or if planning additional pregnancy. Mothers were primarily recruited from lists of children receiving services through the California Department of Developmental Services (a state agency that coordinates services for persons with developmental disabilities). A small percentage of families were also enrolled after being referred by other research studies at the University of California, Davis MIND (Medical Investigation of Neurodevelopmental Disorder) Institute, by other health and service providers who learned about the study at outreach events, or by word of mouth. Inclusion criteria were: (1) mother or father had a biological child with ASD and/or the gestating young child had an older half-sibling, or an equivalent or closer blood relative, with ASD; (2) mother was 18 years or older; (3) mother was pregnant/planning a pregnancy; (4) mother was English proficient and younger siblings would be taught English; (5) the mother was living within 2.5 h of Davis/Sacramento at the time of enrollment. The older sibling's ASD diagnosis was confirmed using the Social-Communication Questionnaire and staff members requested report of prior diagnosis [20].

### Compliance with Ethical Declarations

The UC Davis IRB Administration approved the MARBLES study (IRB ID 225645-74) and informed consent was obtained from each participant. The UC Davis IRB Administration reviewed the objectives and procedure for the supplemental analysis of previously collected BF data from the MARBLES study (IRB ID 1682387+1), and it was determined that IRB review was not required (only de-identified information was utilized).

### Exposure Data

In MARBLES, the mothers completed monthly diaries for the first year and then quarterly diaries thereafter until completion of the study. The purpose of the diaries was to capture rapidly changing exposures or experiences. These monthly and then quarterly diaries collected information about if mothers were continuing to breastfeed at that time and if not when they stopped. Information about ever BF, BF at 3 months, BF at 6 months, BF at 12 months was coded as dichotomous data (yes/no). Months of BF was calculated by subtracting the date of birth from the BF stop date if last date of BF was known, or the last known BF date if stop date was unknown, and then divided by 30.43 (the average number of days in a month) and rounded to two decimal spaces. BF duration was divided into categories: 0 to 3 months, > 3 months to 6 months, > 6 months to 12 months, and > 12 months. These BF durations are consistent with BF categories regularly used by the CDC [9].

### Outcome Data

The child’s developmental assessments conducted in MARBLES included autism related instruments and measures of cognitive ability. Children were regularly assessed after birth until the age of 3 years. The 36-month visit was conducted ± 6 months from when the child turned 3 years old. Development was assessed using the Mullen Scales of Early Learning (MSEL). ASD symptomatology was assessed using the Autism Diagnostic Observation Schedule (ADOS).

MSEL is a comprehensive measure of cognitive function for children from birth to 68 months. Scores from four of the MSEL scales were included in the analysis: visual reception, fine motor, receptive language, and expressive language. The raw scores from each scale were converted to a T score with a mean of 50 and standard deviation of 10. The T scores from the visual reception, fine motor, receptive language, and expressive language are combined and converted to an Early Learning Composite (ECL). The ECL is a standard score with a mean of 100 and a standard deviation of 15 [34]. Trained staff members administer the MSEL.

The ADOS is a semi-structured, standardized assessment used to observe a child’s social interaction, communication, and play skills, as well as repetitive behaviors for children with concern for ASD. There are different modules depending on age and expressive language abilities [30]. The MARBLES study transitioned from the ADOS to the ADOS-2 after the revision was published in 2012. The total score on the ADOS was converted to an ADOS comparison score (ADOScs), also known as severity score, as a measure of severity of autism related symptoms. The comparison score ranges from 1 to 10, with a higher score indicating more severe autism symptoms [18]. The comparison score allows for comparison of symptom severity across modules. Expert clinicians administering the assessments were trained and were research reliable on the ADOS.

Neurodevelopmental outcome classification was determined based on information from the 36-month visit. The outcome algorithm classified children as ASD, TD (typical development), and Non-TD (non-typical development) based on the MSEL and ADOS scores (Table 1). The algorithmically defined outcomes are derived from previously published methods from the Baby Sibling Research Consortium [20, 38].

**Table 1:** Criteria for Neurodevelopmental Outcome Classification at 36-month visit [38]

Neurodevelopmental Outcome Classification	Criteria
Typical Development (TD)	Does not meet criteria for ASD classification *and* No more than 1 MSEL subtest ≥ 1.5 SD below mean *and* No MSEL subtest ≥ 2 SD below mean *and* ADOS > 3 points below ASD cutoff
Autism Spectrum Disorder (ASD)	At or above the ASD cutoff of the ADOS *and* Meet criteria for DSM-5 criteria for ASD
Non-Typical Development (Non-TD)	Does not meet criteria for ASD classification *and* Two or more MSEL subtest ≥ 1.5 SD below mean *and/or* One or more MSEL subtest ≥ 2 SD below mean *and/or* ADOS < 3 points below ADOS cutoff

*MSEL* Mullen Scales of Early Learning, *ADOS* Autism Diagnostic Observation Schedule, *DSM-5* Diagnostic and Statistical Manual of Mental Disorders-5

### Statistical Analysis

All children who had completed the MARBLES study protocol were considered. Exclusion criteria included children outside the age range for the 3-year evaluation for the MSEL or ADOS (36 months ± 6 months), missing MSEL score, or no sibling with ASD.

For both the breastfeeding exposure and the neurodevelopmental outcomes, unadjusted associations with demographic variables were evaluated using Pearson’s chi-square or Fisher’s exact test for categorical variables and Analysis of variance (ANOVA) or the non-parametric Kruskal–Wallis test was used to compare continuous variables, depending on the underlying distribution of the variables. When more than 2 categories were being compared, if the overall test was significant, post-hoc pairwise comparisons, with the Bonferroni correction, or Tukey HSD was conducted to address multiple comparisons.

Similarly, descriptive statistics were used also to characterize the relative frequency of BF exposures and the distribution of duration of BF with the neurodevelopmental outcomes. Pearson’s chi-squared test or Fisher’s exact test were used to compare the percentage of BF. The Kruskal–Wallis test was used to compare median BF duration across neurodevelopmental outcomes. Time to BF cessation for the mothers who initiated BF was compared across the neurodevelopmental outcomes using a log-rank test.

BF exposure was evaluated in regression models. The confounding variables were selected based on previous literature on BF and ASD [1, 8, 24, 45, 48]. Multiple confounder models were considered. Confounder models included: 1- all confounders (sex, gestational age (GA), maternal age, maternal education, homeownership (a proxy for socioeconomic status), insurance status, and marital status), 2—the most parsimonious model for significant confounders for both the exposure and outcome (maternal education, marital status), and 3—no confounders. All three confounder models were used in the analyses listed below. Main reported results are from the 2nd model, though results from other model are found in the supplemental material (Supplemental Tables 2–7).

**Table 2:** Demographic and Clinical Characteristics of Children and their Mothers in the MARBLES Study by Exposure

BF duration categories	Total	0–3 months	> 3–6 months	> 6–12 months	> 12 months	Statistical result
n (%)	308	64 (20.8%)	38 (12.3%)	78 (25.3%)	128 (41.6%)	
Clinical Classification^A^, n (%)						χ^2^(6) = 4.42, *p* = 0.62
TD	198 (64.3%)	39 (60.9%)	23 (60.5%)	51 (65.4%)	85 (66.4%)	
ASD	68 (22.1%)	16 (25.0%)	12 (31.6%)	17 (21.8%)	23 (18.0%)	
Non-TD	42 (13.6%)	9 (14.1%)	3 (7.9%)	10 (12.8%)	20 (15.6%)	
Sex^A^, n (%)						χ^2^(3) = 1.42, *p* = 0.70
Male	174 (56.5%)	36 (56.3%)	20 (52.6%)	41 (52.6%)	77 (60.2%)	
Female	134 (43.5%)	28 (43.8%)	18 (47.4%)	37 (47.4%)	51 (39.8%)	
Mean Gestational Age (Weeks)^B^, mean (SD)	38.9 (1.54)	38.9 (1.67)	39.0 (1.22)	39.0 (1.30)	38.9 (1.69)	χ^2^(3) = 0.38, *p* = 0.95
Child Ethnicity^A^, n (%)						χ^2^(3) = 3.81, *p* = 0.28
Not Hispanic	214 (69.5%)	46 (71.9%)	22 (57.9%)	52 (66.7%)	94 (73.4%)	
Hispanic	94 (30.5%)	18 (28.1%)	16 (42.1%)	26 (33.3%)	34 (26.6%)	
Child Race^C^, n (%)						*p* = 0.69
White	192 (62.3%)	45 (70.3%)	23 (60.5%)	49 (62.8%)	75 (58.6%)	
Black/African American	10 (3.2%)	2 (3.1%)	2 (5.3%)	3 (3.8%)	3 (2.3%)	
Asian	43 (14.0%)	8 (12.5%)	7 (18.4%)	8 (10.3%)	20 (15.6%)	
Other (More than 1 race, American Indian/Alaska Native, Native Hawaiian/Pacific Islander)	63 (20.5%)	9 (14.1%)	6 (15.8%)	18 (23.1%)	30 (23.4%)	
Maternal Education^A^, n (%)						χ^2^(3) = 8.16, *p* = 0.04*
Less than Bachelor’s Degree	149 (48.4%)	35 (54.7%)	24 (63.2%)	39 (50%)	51 (39.8%)	
Bachelor’s/Graduate/Professional degree	159 (51.6%)	29 (45.3%)	14 (36.8%)	39 (50%)	77 (60.2%)	
Maternal Age (Years)^D^, mean (SD)	34.6 (4.85)	34.8 (6.02)	34.2 (5.28)	34.0 (4.56)	35.0 (4.20)	*F*(3,304) = 0.85, *p* = 0.47
Insurance Type^Aa^, n (%)						χ^2^(3) = 2.86, *p* = 0.41
Private	240 (79.2%)	46 (71.9%)	29 (78.4%)	63 (81.8%)	102 (81.6%)	
Public	63 (20.8%)	18 (28.1%)	8 (21.6%)	14 (18.2%)	23 (18.4%)	
Home Ownership^Ab^, n (%)						χ^2^(3) = 7.05, *p* = 0.07
Renter	121 (40.6%)	27 (43.5%)	21 (58.3%)	31 (40.3%)	42 (34.1%)	
Owners	177 (59.4%)	35 (56.5%)	15 (41.7%)	46 (59.7%)	81 (65.9%)	
Marital Status of Mother^Cc^, n (%)						*p* = 0.01*
Married or Living as Married	274 (91.3%)	56 (90.3%)	27 (75%)	72 (93.5%)	119 (95.2%)	
Other (divorced, separated, single, widowed)	26 (8.7%)	6 (9.7%)	9 (25%)	5 (6.5%)	6 (4.8%)	

*BF* breastfeeding, *TD* typical development, *ASD* autism spectrum disorder, *Non-TD* non-typical development, *SD* standard deviation

^*^p value = or < 0.05

Statistical Analysis

^A^Chi-squared test

^B^Kruskal–Wallis rank sum test

^C^Fisher’s exact test

^D^Analysis of variance

Missing Data

^a^Missing data = 5

^b^Missing data = 10

^c^Missing data = 8

Linear regression was used to characterize the association between BF duration and the scores on the MSEL. The distribution of ADOScs was highly skewed with many participants scoring at the lowest value. Therefore, ADOScs was transformed by subtracting one (the lowest score) prior to analysis and then analyzed using negative binomial regression with a log link to characterize the association between BF duration and the ADOScs. Multinominal logistic regression was used to characterize the association between BF duration and neurodevelopmental outcome classification. Participants who had missing confounder data were excluded from these models.

R version 4.0.4 and SAS software version 9.4 (AS Institute Inc., Cary, NC) were used for statistical analysis.

## Results

A total of 325 participants born between December 2006 to January 2017 completed their evaluation at the 36-month visit. 17 were excluded from the study: 6 of the participants were outside the age range for the 36-month visit (assessment was done more than ± 6 months from 3rd birthday) for the MSEL or ADOS, 9 had missing MSEL data, and 2 had no siblings with ASD but were part of a family considered higher risk for ASD. 308 participants remained and were included in the analysis for demographics and comparison of BF duration between neurodevelopmental outcomes. 14 additional participants did not have complete data for confounders and were not included in analysis related to the outcome of MSEL scores, ADOScs scores, and neurodevelopmental outcome classification (hypothesis 1 and 2: n = 294).

In the cohort 56.5% of the children were male. The mean GA (the length of a pregnancy after the first day of the last menstrual period) was 38.9 weeks. 30.5% of the children were Hispanic. A majority of the children were white at 62.3%, while 3.2% were Black/African American, 14.0% were Asian, and 20.5% were other. 48.4% of the mothers had less than a bachelor’s degree (less than high school, high school diploma/GED, some college), while the remainder had a bachelor’s degree or higher. The mean maternal age at the time of delivery was 34.6 years. At the time of delivery, 79.2% had private insurance and the rest had public insurance. For home ownership, 40.6% were renters and the remainder owned their homes. 91.3% classified their marital status as married or living as married (Table 2).

In this enriched-likelihood cohort, 20.8% breastfed for 0–3 months, 12.3% breastfed for > 3–6 months, 25.3% breastfed for > 6–12 months, and 41.6% breastfed for > 12 months. There were statistically significant differences in maternal education and marital status across the BF durations (Table 2). Pairwise comparisons found that for marital status, the > 3–6 months vs. 12 + months of BF were significantly different with a higher percentage married in the 12 + month group (Married: 75% in > 3–6 month vs 95.2% in + 12 month, *p* = 0.001) after accounting for multiple comparisons.

After the 36-month evaluation visit, 64.3% of children were classified as TD, 22.1% were classified as ASD, and 13.6% were classified as Non-TD. There was a statistically significant difference between the neurodevelopmental outcomes for sex, mean GA, maternal education, insurance type, and marital status (Supplemental Table 1). Pairwise comparisons found that for sex the ASD group had significantly more males than the TD group (Male Sex: 72.1% in ASD vs. 52.0% in TD, *p* = 0.006), GA at birth for the ASD group was significantly longer than for the TD group (GA 39.4 (1.13) in ASD vs. 38.8 (1.47) in TD, *p* = 0.01), and significantly fewer mothers were married in the Non-TD group compared to the TD group (Married: 77.5% Non-TD vs. 93.4% TD, *p* = 0.005) after accounting for multiple comparisons.

**Fig. 1:**
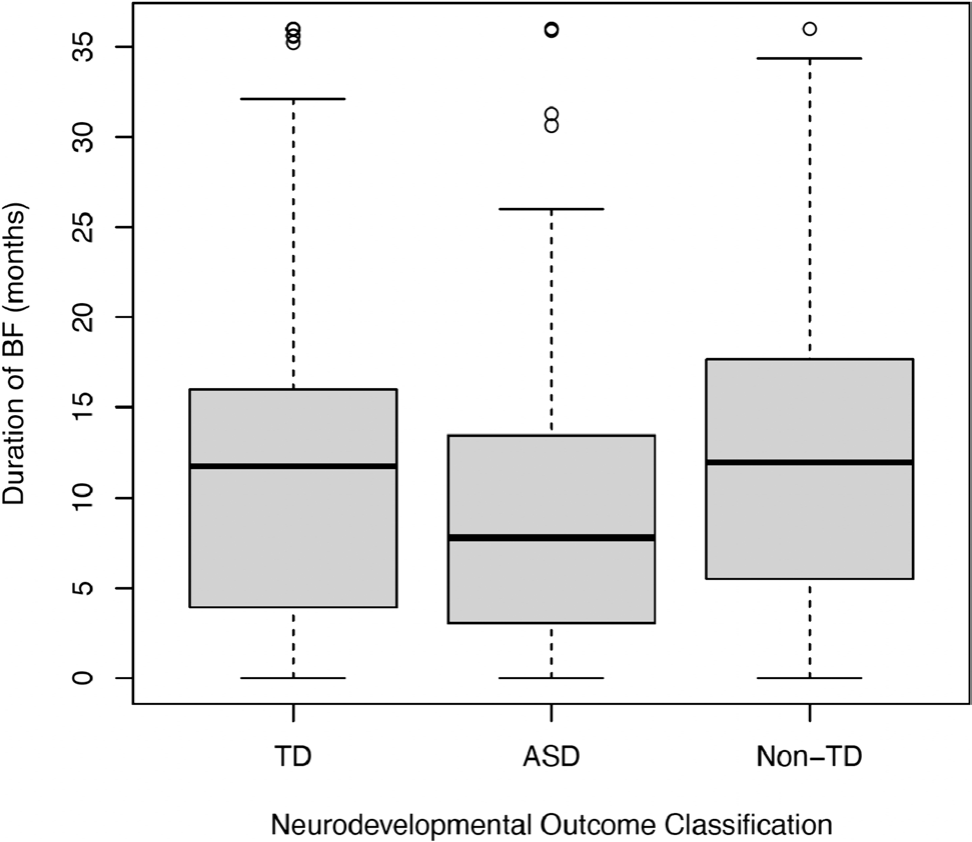
Box plot showing distribution of BF duration across the neurodevelopment outcome classification. Box represents interquartile range (IQR) from first (Q1) to third quartile (Q3), the median is the line across the box. The IQR is Q3-Q1. Whiskers extend to the most extreme data point that is no more than 1.5xIQR from edge of box. Figure generated using R version 4.0.4. *BF* breastfeeding, *TD* typical development, *ASD* autism spectrum disorder, *Non-TD* non-typical development

**Fig. 2:**
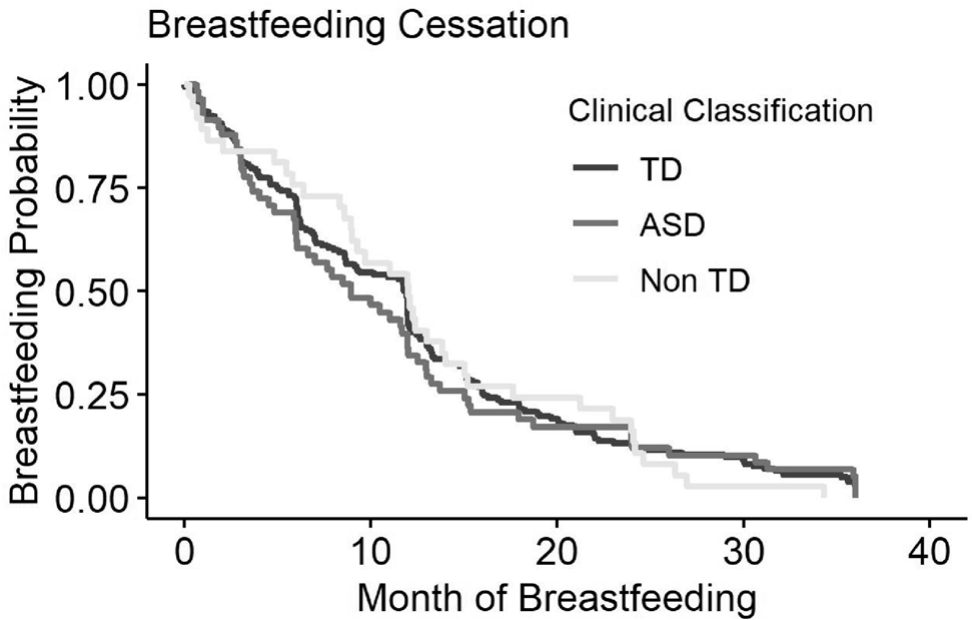
Breastfeeding probability by month for the neurodevelopmental outcome classification. Figure generated in R version 4.0.3. *TD* typical development, *ASD* autism spectrum disorder, *Non-TD* non-typical development

The percentage of ever BF, percentage of BF at 3,6, 12 months, and median duration of BF (Fig. 1) were compared across the neurodevelopmental outcome classification and no statistically significant differences were found (Table 3). The mean duration of BF for all children was 11.70 months (standard deviation (SD) = 9.76), for the TD group was 11.95 months (SD = 9.79), for the ASD group was 10.56 months (SD = 9.84), and for the nonTD group was 12.38 months (SD = 9.55). Kaplan–Meier curves for BF cessation for the different neurodevelopmental outcomes showed no significant difference (n = 277, 17 did not BF; log-rank test; χ^2^(2) = 0.07, p = 0.96; Fig. 2).

**Table 3:** Breastfeeding Characteristics of Children and their Mothers in the MARBLES Study Based on Neurodevelopmental Outcome Classification

	Ever BF^A^, n (%)	BF at 3 months^Ba^, n (%)	BF at 6 months^Bb^, n (%)	BF at 12 months^Bc^, n (%)	Median duration of BF months, Median (IQR)^C^
Total	291 (94.5%)	260 (84.4%)	217 (70.5%)	153 (49.7%)	10.70 (12.07)
TD	188 (94.9%)	170 (85.9%)	142 (71.7%)	102 (51.5%)	11.75 (12.03)
ASD	63 (92.6%)	57 (83.8%)	43 (63.2%)	28 (41.2%)	7.79 (10.28)
Non-TD	40 (95.2%)	33 (78.6%)	32 (76.2%)	23 (54.8%)	11.98 (11.47)
Statistical Result	*p* = 0.71	χ^2^(2) = 0.23, *p* = 0.89	χ^2^(2) = 3.87,* p* = 0.14	χ^2^(2) = 2.99,* p* = 0.22	χ^2^(2) = 2.04, p = 0.36

^*^p value = or < 0.05

*BF* breastfeeding, *IQR* interquartile range, *TD* typical development, *ASD* autism spectrum disorder, *Non-TD* non-typical development

Statistical Analysis

^A^Fisher’s exact test

^B^Chi-squared test

^C^Kruskal–Wallis rank sum test

Missing Data

^a^Missing data = 9

^b^Missing data = 12

^c^Missing data = 32

In regards to hypothesis 1, the models comparing MSEL scale scores and ECL score with the BF duration categories, there was no significant overall group difference. However, MSEL fine motor scale score, receptive language scale score, expressive language scale scores, and ECL score were significantly higher in children who were breastfed > 12 months compared to children who were breastfed for 0–3 months (Table 4). When BF duration was analyzed in relation to ADOScs, which is used to indicate ASD severity, the direction of association was in the expected negative direction (reduced severity with longer duration) but was not statistically significant (χ^2^(3) = 1.83, p = 0.61; Table 5).

**Table 4:** Associations of Breastfeeding Duration Categories and MSEL Scores (n = 294)

	0–3 months	> 3–6 months	> 6–12 months	> 12 months	Statistical Result
Visual reception					F(3, 288) = 0.87, *p* = 0.46
Estimated coefficient (95% CI)	REF	– 2.43 (– 8.71 to 3.84)	0.28 (– 5.01 to 5.57)	2.01 (– 2.68 to 6.69)	
p-value^a^	REF	0.45	0.92	0.40	
Fine motor					F(3, 288) = 1.64, *p* = 0.18
Estimated coefficient (95% CI)	REF	3.85 (– 2.14 to 9.85)	3.79 (– 1.27 to 8.85)	5.03 (0.54 to 9.51)	
p-value^a^	REF	0.21	0.14	0.03*	
Receptive language					F(3, 288) = 1.93, *p* = 0.13
Estimated coefficient (95% CI)	REF	0.17 (– 4.32 to 4.67)	3.15 (– 0.64 to 6.94)	3.44 (0.08 to 6.80)	
p-value^a^	REF	0.94	0.10	0.04*	
Expressive language					F(3, 288) = 1.75, *p* = 0.16
Estimated coefficient (95% CI)	REF	1.75 (– 3.11 to 6.60)	4.08 (– 0.01 to 8.17)	3.73 (0.10 to 7.35)	
p-value^a^	REF	0.48	0.05	0.04*	
Early learning composite					F(3, 288) = 1.58, *p* = 0.20
Estimated coefficient (95% CI)	REF	1.57 (– 7.26 to 10.40)	5.32 (– 2.12 to 12.76)	6.71 (0.12 to 13.31)	
p-value^a^	REF	0.73	0.16	0.05*	

The model includes maternal education and marital status as confounders

*MSEL* Mullen Scales of Early Learning, *CI* confidence interval

^*^p-value = or < 0.05

^a^0–3 months as reference group

**Table 5:** Association of Breastfeeding Duration Categories and ADOS Comparison Score (n = 294)

	0–3 months	> 3–6 months	> 6–12 months	> 12 months	Statistical Result
ADOScs					χ^2^(3) = 1.83, *p* = 0.61
Estimated coefficient (95% CI)	REF	– 0.06 (– 0.83 to 0.71)	– 0.19 (– 0.82 to 0.45)	– 0.36 (– 0.92 to 0.21)	
p-value^a^	REF	0.88	0.56	0.21	

The model includes maternal education and marital status as confounders

*ADOScs* Autism Diagnostic Observation Schedule comparison score

CI = confidence interval

^*^p-value = or < 0.05

^a^0–3 months as reference group

Counter to Hypothesis 2, there were no statistically significant associations between longer BF duration and lower odds of ASD or nonTD (χ^2^(6) = 5.19, p = 0.52; Table 6).

**Table 6:** Associations of Breastfeeding Duration Categories and Neurodevelopmental Outcome Classification (n = 294)

		0–3 months	> 3–6 months	> 6–12 months	> 12 months	Statistical Result
	Neurodevelopmental outcome classification					χ^2^(6) = 5.19, *p* = 0.52
	ASD vs. TD					
	Odds Ratio (95%CI)	REF	1.25 (0.48–3.26)	1.01 (0.43–2.35)	0.73 (0.33–1.59)	
	p-value^a^	REF	0.43	0.91	0.20	
	Non-TD vs. TD					
	Odds Ratio, 95%CI	REF	0.44 (0.10–1.97)	0.93 (0.31–2.80)	1.31 (0.51–3.37)	
	p-value^a^	REF	0.20	0.83	0.15	

The model includes maternal education and marital status as confounders

*CI* confidence interval, *TD* typical development, *ASD* autism spectrum disorder, *Non-TD* non-typical development

^*^p-value = or < 0.05

^a^0–3 months as reference group

## Discussion

In this enriched-likelihood cohort, we investigated differences in duration of BF for children with ASD compared to the other neurodevelopmental outcomes, and whether neurodevelopmental assessment results and neurodevelopmental outcome classification differed based on BF duration. Children who were classified as ASD, TD, or Non-TD had statistically similar rates of ever BF, BF at 3, 6, and 12 months. While the median duration of BF in the ASD group (7.79 months) was less than the TD (11.75 months) and Non-TD group (11.98 months), the difference in distribution was not significant (χ^2^(2) = 2.04, p = 0.36).

When evaluating neurodevelopmental assessment scores and the neurodevelopmental outcomes in this enriched-likelihood cohort, BF for > 12 months was associated with statistically significantly higher scores on the MSEL (fine motor, receptive language, expressive language, and ECL score) compared to children who BF for 0–3 months. We did not find an association between longer BF duration and the lower ADOScs or ASD risk.

We did not find significant differences in the percentage of BF at 3, 6, or 12 months or median duration of BF when comparing the TD, ASD, and Non-TD groups. To our knowledge this is the first time this relationship was investigated prospectively in an enriched-likelihood cohort for ASD. Our result was similar to Emond et al. [15] and Nadon et al. [35] who found no difference in rates of BF for children with ASD when compared to the control group. Our results differ from other studies which have found differences in rates and duration of BF for children with ASD [16, 29, 45, 48]. We may not have found significant results due to the small sample size of our study. Future studies of enriched-likelihood cohorts for ASD should consider continuing to investigate the relationship with BF duration, since a larger sample size will help to clarify if there is a relationship between shorter BF duration and ASD diagnosis in this population.

We did find improved scores on tests of cognitive ability (MSEL) with longer duration of BF in our cohort. These findings are consistent with other studies in the field which have found cognitive benefit to BF [3, 14, 21, 22, 28, 50]. Some proposed mediators for the impact of BF on cognitive development include differences in parent–child interaction during BF, nutritional differences of breast milk, or non-nutritive bioactive factors present in breast milk [5]. There are also structural differences in brain development that are seen in MRI studies, such as increased white matter development [12] or increased cortical thickness in parietal lobe ([25] for children who are BF; these structural differences could impact cognitive development.

Finally, we did not find an association between BF duration and ASD symptomatology or risk of ASD classification. This is consistent with [24], where ASD was not associated with BF history. This does differ from other studies that suggest BF duration influences ASD risk [1, 7, 8, 17, 23, 43, 44]. This discrepancy could be since our study included children with an enriched-likelihood for ASD, so they inherently have a higher background risk for ASD compared to the general population [20, 40]. As a result, BF may not have a similar relationship to ASD in this cohort as seen in prior studies.

Strengths of the study include that the data were prospectively collected, eliminating the concern for recall bias. Additionally, the neurodevelopmental assessments were performed using standardized assessments by trained clinicians, increasing the validity of these results. Finally, the comprehensive data collection through MARBLES allowed us to adjust for a variety of confounding variables. This study also adds to this field of literature since it is the first time the relationship between ASD and BF has been studied prospectively in an enriched-likelihood cohort.

A limitation of our study is that the cohort is not representative of the general population due to the inherent increased background risk for ASD, so the results are not generalizable to the population as a whole. We also had a small sample size which may have underpowered our ability to find a significant association between BF and ASD risk. We also acknowledge that while ASD diagnosis at 3 years of age is reliable and stable [41], a small portion (< 3%) of children not diagnosed with ASD at age 3 years will develop symptoms and meet criteria for diagnosis at older ages [39], thus this study does not account for children in our cohort who receive a later diagnosis. Additionally, we were not able to assess if and how long the participants were exclusively BF. Future studies examining exclusive BF or evaluating the intensity of BF, which is the percentage of all feedings that are breast milk [36], could allow a more careful dose–response analysis of the relationship between BF and ASD.

Finally, we acknowledge that terminology used to describe both ASD and BF are evolving over time. The terminology used in this article was selected with the goal of clarity and accuracy to how the original MARBLES study collected data. We recognize some terminology used may not be affirming for all people and families in this study. Also, the MARBLES study did not ask the gender of the birthing parent and presumed gender based on sex. We recognize that this may not represent the experience of gender diverse parents included in this cohort.

These findings are relevant for parents who have a child with ASD. Longer duration of BF could be associated with improved cognitive development for subsequent children. However, our findings do not support BF as a way to decrease ASD symptomatology or ASD risk for their future children. This is an important message to these parents since their choice on how to feed their infant might not impact their risk of developing ASD, which could alleviate a parent’s stress or feelings of guilt surrounding feeding choices. Despite not finding an association between BF and ASD risk in this enriched-likelihood cohort, BF should continue to be encouraged to interested parents for improved cognitive development, along with the variety of other health benefits BF has for both the infant and parent [33].

This topic remains important for continued research because decreasing risk for ASD and identifying factors that improve cognitive ability for children who have an enriched-likelihood for ASD has clinical significance. Future studies evaluating if there is a dose–response relationship between BF intensity and ASD risk in an enriched-likelihood cohort can provide information on whether the amount of breast milk consumed impacts risk for ASD. Additionally, studies following enriched-likelihood cohorts for ASD should consider monitoring BF behavior prospectively to see if suboptimal BF practices, such as dysregulated BF or difficulty with weaning from BF [49], could be an early indicator for ASD or developmental delay.

## Summary

In this enriched-likelihood cohort, there was no statistically significant difference in duration of BF based on the neurodevelopmental outcome classification. For children in an enriched-likelihood cohort for ASD, BF for a longer duration was associated with improved cognitive ability at 3 years of age; however, BF duration was not associated with decreased ASD symptomatology or decreased ASD risk. Additional larger studies investigating intensity of BF or challenges in BF for children at risk for ASD are needed.

## Supplementary Information

Below is the link to the electronic supplementary material.

Supplementary file1 (DOCX 44 KB)

## Data Availability

Data from the MARBLES study is deposited in the National Database for Autism Research, which is publicly available. https://nda.nih.gov/edit_collection.html?id=2557
