# Supplementary material for: Association of Breastfeeding Duration with Neurodevelopmental Outcomes in an Enriched Familial Likelihood Cohort for Autism Spectrum Disorder
Source: Child Psychiatry Hum Dev. 2024 Apr 24;57(1):188–99. doi: 10.1007/s10578-024-01700-7 (PMC11499290; doi:10.1007/s10578-024-01700-7)
Supplement: Supplementary file 1 — Supplementary file1 (DOCX 44 KB) [file 10578_2024_1700_MOESM1_ESM.docx]

**Title:**

Association of Breastfeeding Duration with Neurodevelopmental Outcomes in an Enriched Familial Likelihood Cohort for Autism Spectrum Disorder

**Journal:**

Child Psychiatry & Human Development

**Authors:**

Ruchi Punatar^1,2^, Kathleen Angkustsiri^1,2^, Laura R. Kair^3^, Daniel J. Tancredi^3^, Danielle J. Harvey^4^, Rebecca J. Schmidt^2,4, *^

**Institutions:**

^1^ University of California Davis, Department of Pediatrics, Division of Developmental Behavioral Pediatrics, Sacramento, California, USA

^2^ University of California Davis, UC Davis MIND (Medical Investigation of Neurodevelopmental Disorders) Institute, Sacramento, California, USA

^3^ University of California Davis, Department of Pediatrics, Sacramento, California, USA

^4^ University of California Davis, Department of Public Health Sciences, Davis, California, USA

^*^ Corresponding Author

**Corresponding author contact information:**

e-mail: rjschmidt@ucdavis.edu

phone: 530-752-3226

fax: 520-752-3239

Address:

University of California, Davis

One Shields Avenue, Med Sci 1C

Davis, CA 95616

**Supplemental Information**

Supplemental Table 1: Demographic and Clinical Characteristics of Children and their Mothers in the MARBLES Study by Outcome

| Clinical Classification | | Total | TD | ASD | Non-TD | Statistical Result |
| --- | --- | --- | --- | --- | --- | --- |
| n (%) | | 308 | 198 (64.3%) | 68 (22.1%) | 42 (13.6%) |  |
| BF Duration Categories^A^   n (%) | | | | | | 𝜒^2^(6)=4.42, *p*=0.62 |
|  | 0-3 months | 64 (20.8%) | 39 (19.7%) | 16 (23.5%) | 9 (21.4%) |  |
|  | >3-6 months | 38 (12.3%) | 23 (11.6%) | 12 (17.6%) | 3 (7.1%) |  |
|  | >6-12 months | 78 (25.3%) | 51 (25.8%) | 17 (25.0%) | 10 (23.8%) |  |
|  | >12 months | 128 (41.6%) | 85 (42.9%) | 23 (33.8%) | 20 (47.6%) |  |
| Sex^A^  n (%) | | | | | | 𝜒^2^(2)=8.60, *p*=0.01* |
|  | Male | 174 (56.5%) | 103 (52.0%) | 49 (72.1%) | 22 (52.4%) |  |
|  | Female | 134 (43.5%) | 95 (48.0%) | 19 (27.9%) | 20 (47.6%) |  |
| Mean Gestational Age (Weeks)^B^  mean (SD) | | 38.9 (1.54) | 38.8 (1.47) | 39.4 (1.13) | 38.7 (2.14) | 𝜒^2^(2)=10.34, *p*=0.01* |
| Child Ethnicity^A^  n (%) | | | | | | 𝜒^2^(2)=0.41, *p*=0.82 |
|  | Not Hispanic | 214 (69.5%) | 140 (70.7%) | 46 (67.6%) | 28 (66.7%) |  |
|  | Hispanic | 94 (30.5%) | 58 (29.3%) | 22 (32.4%) | 14 (33.3%) |  |
| Child Race^C^  n (%) | | | | | | *p*=0.06 |
|  | White | 192 (62.3%) | 126 (63.6%) | 40 (58.8%) | 26 (61.9%) |  |
|  | Black/African American | 10 (3.2%) | 2 (1.0%) | 7 (10.3%) | 1 (2.4%) |  |
|  | Asian | 43 (14.0%) | 28 (14.1%) | 9 (13.2%) | 6 (14.3%) |  |
|  | Other (More than 1 race, American Indian/Alaska Native, Native Hawaiian/Pacific Islander) | 63 (20.5%) | 42 (21.2%) | 12 (17.6%) | 9 (21.4%) |  |
| Maternal Education^A^  n (%) | | | | | | 𝜒^2^(2)=6.62, *p*=0.04* |
|  | Less than Bachelor’s Degree | 149 (48.4%) | 85 (42.9%) | 40 (58.8%) | 24 (57.1%) |  |
|  | Bachelor’s/  Graduate/  Professional  Degree | 159 (51.6%) | 113 (57.1%) | 28 (41.2%) | 18 (42.9%) |  |
| Maternal Age (Years)^D^  mean (SD) | | 34.6 (4.85) | 34.8 (4.91) | 34.5 (4.93) | 33.7 (4.43) | F(2,305)=0.91, *p=*0.40 |
| Insurance Type^Aa^ | | | | | | 𝜒^2^(2)=9.38, *p*=0.01* |
|  | Private | 240 (79.2%) | 164 (84.5%) | 48 (70.6%) | 28 (68.3%) |  |
|  | Public | 63 (20.8%) | 30 (15.5%) | 20 (29.4%) | 13 (31.7%) |  |
| Home Ownership^Ab^  n (%) | | | | | | 𝜒^2^(2)=3.87, *p*=0.14 |
|  | Renter | 121 (40.6%) | 72 (36.9%) | 32 (50.8%) | 17 (42.5%) |  |
|  | Owners | 177 (59.4%) | 123 (63.1%) | 31 (49.2%) | 23 (57.5%) |  |
| Marital Status of Mother^Cc^  n (%) | | | | | | *p*=0.01* |
|  | Married or Living as Married | 274 (91.3%) | 183 (93.4%) | 60 (93.8%) | 31 (77.5%) |  |
|  | Other (divorced, separated, single, widowed) | 26 (8.7%) | 13 (6.6%) | 4 (6.3%) | 9 (22.5%) |  |

*p value = or <0.05

TD = typical development, ASD = autism spectrum disorder, Non-TD = non-typical development, BF = breastfeeding, SD = standard deviation

Statistical Analysis

A: Chi-squared test

B: Kruskal-Wallis rank sum test

C: Fisher’s exact test

D: Analysis of variance

Missing Data:

a: missing data =5

b: missing data = 10

c: missing data =8

Supplemental Table 2: Association of Breastfeeding Duration Categories and MSEL Scores (n=294)

|  |  | 0-3  months | >3-6 months | >6-12 months | >12 months | Statistical Result |
| --- | --- | --- | --- | --- | --- | --- |
| Visual Reception |  |  |  |  |  | F(3,283)=0.97  *p=*0.41 |
|  | Estimated  coefficient  (95% CI) | REF | -2.77  (-8.96 to 3.43) | -0.27  (-5.54 to 4.99) | 1.86  (-2.78 to 6.50) |  |
|  |  |  |  |  |  |  |
|  | p-value^a^ | REF | 0.38 | 0.92 | 0.43 |  |
| Fine Motor |  |  |  |  |  | F(3,283)=1.59  *p=*0.19 |
|  | Estimated  coefficient  (95% CI) | REF | 3.56  (-2.39 to 9.50) | 2.78  (-2.28 to 7.83) | 4.88  (0.43 to 9.34) |  |
|  | p-value^a^ | REF | 0.24 | 0.28 | 0.03* |  |
| Receptive Language |  |  |  |  |  | F(3,283)=1.75  *p=*0.16 |
|  | Estimated  coefficient  (95% CI) | REF | -0.13  (-4.53 to 4.26) | 2.39  (-1.35 to 6.13) | 3.25  (-0.05 to 6.54) |  |
|  | p-value^a^ | REF | 0.95 | 0.21 | 0.05 |  |
| Expressive Language |  |  |  |  |  | F(3,28)=1.58  *p=*0.19 |
|  | Estimated  coefficient  (95%CI) | REF | 1.32  (-3.45 to 6.10) | 3.38  (-0.68 to 7.44) | 3.66  (0.08 to 7.23) |  |
|  |  |  |  |  |  |  |
|  | p-value^a^ | REF | 0.59 | 0.10 | 0.05* |  |
| Early Learning Composite |  |  |  |  |  | F(3,283)=1.52  *p=*0.21 |
|  | Estimated  coefficient  (95% CI) | REF | 0.93  (-7.73 to 9.60) | 3.92  (-3.44 to 11.29) | 6.45  (-0.04 to 12.94) |  |
|  | p-value^a^ | REF | 0.83 | 0.30 | 0.05 |  |

The model includes sex, gestational age, maternal age, maternal education, homeownership, insurance status, and marital status as confounders.

* p-value = or < 0.05

a: 0-3 months as reference group

MSEL = Mullen Scales of Early Learning

CI = confidence interval

Supplemental Table 3: Association of Breastfeeding Duration Categories and MSEL Scores (n=294)

|  |  | 0-3  months | >3-6  months | >6-12 months | >12 months | Statistical Result |
| --- | --- | --- | --- | --- | --- | --- |
| Visual Reception |  |  |  |  |  | F(3,290)=1.55  *p=*0.20 |
|  | Estimated  coefficient  (95% CI) | REF | -3.16  (-9.55 to 3.23) | 0.62  (-4.80 to 6.03) | 2.78  (-1.99 to 7.56) |  |
|  |  |  |  |  |  |  |
|  | p-value^a^ | REF | 0.33 | 0.83 | 0.25 |  |
| Fine Motor |  |  |  |  |  | F(3,290)=2.06  *p=*0.11 |
|  | Estimated coefficient  (95% CI) | REF | 3.15  (-2.89 to 9.18) | 4.04  (-1.07 to 9.15) | 5.66  (1.14 to 10.17) |  |
|  |  |  |  |  |  |  |
|  | p-value^a^ | REF | 0.31 | 0.12 | 0.01* |  |
| Receptive Language |  |  |  |  |  | F(3,290)=3.04  *p=*0.03* |
|  | Estimated coefficient  (95% CI) | REF | -0.60  (-5.26 to 4.05) | 3.47  (-0.47 to 7.41) | 4.20  (0.72 to 7.68) |  |
|  |  |  |  |  |  |  |
|  | p-value^a^ | REF | 0.80 | 0.08 | 0.02* |  |
| Expressive Language |  |  |  |  |  | F(3,290)=2.36  *p=*0.07 |
|  | Estimated coefficient  (95%CI) | REF | 1.15  (-3.82 to 6.12) | 4.36  (0.15 to 8.57) | 4.37  (0.66 to 8.09) |  |
|  |  |  |  |  |  |  |
|  | p-value^a^ | REF | 0.65 | 0.04* | 0.02* |  |
| Early Learning Composite |  |  |  |  |  | F(3,290)=2.45  *p=*0.06 |
|  | Estimated coefficient  (95% CI) | REF | 0.26  (-8.81 to 9.32) | 5.88  (-1.80 to 13.56) | 8.03  (1.25 to 14.80) |  |
|  | p-value^a^ | REF | 0.96 | 0.13 | 0.02* |  |

The model includes no confounders.

* p-value = or < 0.05

a: 0-3 months as reference group

MSEL = Mullen Scales of Early Learning

CI = confidence interval

Supplemental Table 4. Association of Breastfeeding Duration Categories and ADOS Comparison Score (n=294)

|  |  | 0-3 months | >3-6 months | >6-12 months | >12 months | Statistical Result |
| --- | --- | --- | --- | --- | --- | --- |
| ADOScs |  |  |  |  |  | χ^2^(3)=1.38 *p=*0.71 |
|  | Estimated  coefficient  (95% CI ) | REF | -0.10  (-0.85 to 0.65) | -0.06  (-0.69 to 0.56) | -0.30  (-0.87 to 0.26) |  |
|  |  |  |  |  |  |  |
|  | p-value^a^ | REF | 0.79 | 0.84 | 0.29 |  |

The model includes sex, gestational age, maternal age, maternal education, homeownership, insurance status, and marital status as confounders.

* p-value = or < 0.05

a: 0-3 months as reference group

ADOScs = Autism Diagnostic Observation Schedule comparison score

CI = confidence interval

Supplemental Table 5. Association of Breastfeeding Duration Categories and ADOS Comparison Score (n=294)

|  |  | 0-3 months | >3-6 months | >6-12 months | >12 months | Statistical Result |
| --- | --- | --- | --- | --- | --- | --- |
| ADOScs |  |  |  |  |  | χ^2^(3)=1.56 *p=*0.67 |
|  | Estimated  coefficient  (95% CI) | REF | -0.11  (-0.86 to 0.65) | -0.17  (-0.81 to 0.47) | -0.34  (-0.91 to 0.22) |  |
|  | p-value^a^ | REF | 0.78 | 0.60 | 0.24 |  |

The model includes no confounders.

* p-value = or < 0.05

a: 0-3 months as reference group

ADOScs = Autism Diagnostic Observation Schedule comparison score

CI = confidence interval

Supplemental Table 6. Association of Breastfeeding Duration Categories and Neurodevelopmental Outcome Classification (n=294)

|  |  | 0-3  months | >3-6 months | >6-12 months | >12 months | Statistical Result |
| --- | --- | --- | --- | --- | --- | --- |
| Neurodevelopmental Outcome Classification |  |  |  |  |  | χ^2^(6)=4.90  *p=*0.56 |
| ASD vs. TD | Odds Ratio  (95%CI) | REF | 1.33  (0.49 to 3.64) | 1.15  (0.47 to 2.81) | 0.72  (0.32 to 1.63) |  |
|  | p-value^a^ | REF | 0.43 | 0.67 | 0.15 |  |
| Non-TD vs TD | Odds Ratio  (95%CI) | REF | 0.50  (0.11 to 2.20) | 0.99  (0.32 to 3.01) | 1.27  (0.49 to 3.29) |  |
|  | p-value^a^ | REF | 0.26 | 0.77 | 0.23 |  |

The models includes sex, gestational age, maternal age, maternal education, homeownership, insurance status, and marital status as confounders.

* p-value = or < 0.05

a: 0-3 months as reference group

CI = confidence interval

TD = typical development, ASD = autism spectrum disorder, Non-TD = non-typical development

Supplemental Table 7. Association of Breastfeeding Duration Categories and Neurodevelopmental Outcome Classification (n=294)

|  |  | 0-3 months | >3-6 months | >6-12 months | >12 months | Statistical Result |
| --- | --- | --- | --- | --- | --- | --- |
| Neurodevelopmental Outcome Classification |  |  |  |  |  | χ^2^(6)=4.03  0.67 |
| ASD vs. TD | Odds Ratio  (95%CI) | REF | 1.30  (0.51 to 3.34) | 0.99  (0.43 to 2.28) | 0.69  (0.32 to 1.48) |  |
|  | p-value^a^ | REF | 0.33 | 0.94 | 0.13 |  |
| Non-TD vs TD | Odds Ratio  (95%CI) | REF | 0.62  (0.15 to 2.57) | 0.86  (0.30 to 2.52) | 1.09  (0.44 to 2.70) |  |
|  | p-value^a^ | REF | 0.48 | 0.97 | 0.43 |  |

The model includes no confounders.

* p-value = or < 0.05

a: 0-3 months as reference group

CI = confidence interval

TD = typical development, ASD = autism spectrum disorder, Non-TD = non-typical development
